# Length and Framing of Anti‐Junk Food Ads Impact Inclinations to Consume Junk Food Among Normal Weight, Overweight, and Adults With Obesity

**DOI:** 10.1002/hpja.70159

**Published:** 2026-02-02

**Authors:** Ross C. Hollett, Brennen Mills, Stephanie L. Godrich, Julia Butt, Gina S. A. Trapp

**Affiliations:** ^1^ Psychology & Criminology Edith Cowan University Joondalup Western Australia Australia; ^2^ School of Medical & Health Sciences Edith Cowan University Joondalup and Bunbury Western Australia Australia; ^3^ Nutrition & Health Innovation Research Institute Edith Cowan University Joondalup and Bunbury Western Australia Australia; ^4^ The Kids Institute, The University of Western Australia Perth Western Australia Australia; ^5^ School of Medicine, The University of Western Australia Perth Western Australia Australia

## Abstract

**Background:**

Unrestricted junk food advertising increases the risk of short‐term junk food consumption among viewers. We aimed to estimate the impact of junk food and anti‐junk food advertisements differing in length and framing on junk food consumption inclinations.

**Methods:**

Adult participants (*N* = 505) were exposed to a randomly selected junk food advertisement or anti‐junk food advertisement and reported their immediate craving and consumption intentions. These responses were separately analysed for two groups based on Body Mass Index (BMI): participants classified as within the normal range (18.5–25), and a combined group with BMI values indicating either overweight (25+) or obesity (30+) (OW/OB). A secondary analysis was also performed on a subgroup (*N* = 99) who were exposed to an advertisement containing junk food they reportedly enjoy consuming.

**Results:**

For both BMI groups, junk food advertisement exposure did not increase immediate craving or consumption intentions. However, decreases were observed in craving and consumption intentions following anti‐junk food advertisements. A 15‐s anti‐junk advertisement was more effective than a 30‐s anti‐junk advertisement for normal weight BMI participants. For OW/OB BMI participants, an anti‐junk advertisement encouraging health food was more effective than an anti‐junk advertisement criticising junk food.

**Conclusions:**

The effectiveness of anti‐junk food advertisements varies depending on the length and framing of the advertisements as well as the viewer's BMI categorisation. These nuances are important for maximising the effectiveness of anti‐junk food advertisements in different contexts.

**So What?:**

Given the potential for anti‐junk food advertisements to curb consumption, a higher frequency of broadcasting brief positively framed health messages should be considered to mitigate the potential public health risks associated with junk food consumption.

## 1 Introduction

Junk food (i.e., energy dense/nutrient‐poor food) advertising within Australia and its contribution to unhealthy eating among viewers has attracted considerable concern within the community and among public health experts [1, 2, 3, 4]. Junk food advertisements in Australia remain largely unregulated [5, 6], despite advertising restrictions on other public health risks like gambling and alcohol. While there is clear evidence of a significant effect of junk food advertising exposure on greater food intake in children, studies on adults have produced mixed results [7, 8, 9]. In contrast, anti‐junk food advertisements have been shown to successfully reduce junk food inclinations among both normal and overweight adults [8]. To better understand the risks associated with junk food advertising and benefits of anti‐junk food advertising, it is necessary to further explore the individual differences of audiences as well as the framing of the advertisements themselves. There are several factors which may increase the likelihood an individual will exhibit an inclinational response to a junk food or anti‐junk food advertisement. Firstly, individuals who have a habit of eating junk foods and are classified according to BMI guidelines as overweight or having obesity may be more susceptible than individuals who have a habit of eating healthy foods and are classified as belonging to a normal BMI category [8, 10, 11]. Secondly, when participants are exposed to a product type they have a preference for, they may be more likely to respond to junk food advertisements with increased consumption inclinations, an effect observed in similar research on alcohol advertisements [12].

Length and framing of advertisements may also impact consumption inclinations. For instance, some evidence suggests that the intended message of longer advertisements (e.g., 30 s) is more likely to be later recalled by viewers; however, shorter advertisements (e.g., 15 s) might be more positively received due to their brevity and lower cognitive commitment [13, 14, 15]. As it is not clear in the junk and anti‐junk food context whether shorter advertisements are as effective as longer advertisements, research on the effectiveness of advertisement length could inform public health expenditure. Importantly, advertisements which frame junk or healthy foods unfavourably and favourably (respectively) may differ in their effectiveness for promoting healthy eating. While junk food advertisements will inevitably frame junk food favourably, anti‐junk food advertisements can either focus on criticising junk food or encouraging healthy food alternatives [16]. Some prior research has explored the benefits of negatively framed (e.g., critical) junk food advertisements for attention capture and risk perception, relative to positively framed (e.g., encouraging) advertisements [17, 18]. However, the immediate impact of different anti‐junk food message framing on consumption inclinations would benefit from further investigation, particularly given that there have been mixed results. Specifically, while some research suggests that negative framing is more effective when examining short term responses to food health messages [19], recent research has also found no impact of positive versus negative framing for recall of health messaging [20]. Furthermore, many recent studies have focused on static stimuli, rather than dynamic stimuli, such as the video advertising often embedded into video‐on‐demand and free‐to‐air services [21, 22]. To better inform evidenced‐based advertising standards in Australia and effective health messaging, experimental evidence is needed to determine the varying impact of anti‐junk food advertising length and framing on the reduction of inclinations to consume junk food.

The present study aimed to (1) determine whether junk food advertisements increased, and anti‐junk food advertisements decreased, consumption inclinations among Australian adults who differ in their BMI status, (2) determine if participants with junk food preferences aligning with the advertising content experience stronger craving and consumption intention responses, and (3) explore the extent to which length and framing of junk and anti‐junk food advertisements might impact junk food consumption inclinations. We also explored correlations between responses to junk and anti‐junk food advertisements with suboptimal eating patterns, impulsivity and self‐control to better understand who are most responsive to these advertisements. Note that aims 1 and 3 were previously investigated in a recent study [8], however, the present study extends on this work by including three new experimental conditions (a neutral comparison condition and two additional anti‐junk advertisements) as well as a secondary subgroup analysis not previously performed. As such, the present study functions both as a replication of, and extension to, recent findings but in a new sample of Australian adults. Consistent with the literature and our assumptions, the following hypotheses were tested:

Hypothesis 1 *(a) Junk food craving and (b) consumption intentions would increase following exposure to a single junk food advertisement, with these effects expected to be (c) larger for overweight and those with obesity compared to normal weight participants, according to BMI categories*.

Hypothesis 2 *(a) Junk food craving and (b) consumption intentions would decrease following exposure to a single anti‐junk food advertisement, with these effects expected to be (c) larger for normal weight compared to overweight participants and those with obesity, according to BMI categories*.

Hypothesis 3 *Participants in both BMI groups who are exposed to an advertisement containing junk food that aligns with their current junk food preferences would show increases in (a) craving and (b) consumption intentions*.

Hypothesis 4 *(a) Healthy eating habits, (b) unhealthy eating habits, (c) self‐control and (d) impulsivity measures would negatively (a & c) and positively (b & d) correlate more strongly with post‐exposure junk food craving and consumption intention ratings than pre‐exposure ratings*.

To explore the potential impact of length and framing of the advertisements on craving and consumption inclinations, junk and anti‐junk food advertisements of different lengths (i.e., 15 and 30 s) were analysed separately and anti‐junk food advertisements adopting different framing methods were analysed separately.

## 2 Method

### 2.1 Participants

Australian adults were recruited for this study via a survey deployed to Western Australian University undergraduate psychology students (*N* = 318; 63%) and a Qualtrics panel (*N* = 187; 37%).^1^ The final sample (*N* = 505) excluded incomplete responses and attention check failures (*N* = 26). Specifically, participants were included in the study if they were aged 18 years or older and excluded if they failed the online attention checks.

### 2.2 Materials

#### 2.2.1 Junk Food Craving and Consumption Intentions

A brief version of the state Food Craving Questionnaire (FCQ) [23] estimated junk food craving. Participants rated five statements (e.g., I have an urge for junk food), using a 5‐point scale from 1 (*strongly disagree*) to 5 (*strongly agree*). Item responses were averaged to calculate a total score. The FCQ demonstrated good internal consistency (*α* = 0.94). Junk food consumption intentions were measured using the item ‘I intend to eat junk food as soon as I get the chance’, rated from 1 (*strongly disagree*) to 5 (*strongly agree*). Junk food was defined for participants according to Australian Government [24] criteria, specifically ‘When we refer to “junk food” in this survey, we are referring to foods which are high in energy but low in nutritional value. Examples include sugary drinks, burgers, pizzas, fried chicken and chips/fries’. While exact thresholds can vary, nutritional value generally refers to the quantity of nutrients (e.g., vitamins and minerals) *relative to* energy (e.g., salts, sugars or fats) [25].

#### 2.2.2 Healthy and Unhealthy Eating Scale and Junk Food Preferences

The Healthy and Unhealthy Eating Behaviour Scale (HUEBS; [26]) estimated healthy and unhealthy eating habits as separate subscales. There were 11 healthy eating (e.g., I eat fruits) and 10 unhealthy eating (e.g., I eat fast food) items rated from 1 (*never*) to 7 (*always*).^2^ Item responses were averaged to calculate a total score for healthy and unhealthy eating separately. The HUEBS demonstrated good internal consistency (healthy *α* = 0.81; unhealthy eating *α* = 0.86). Participants were also presented with several categories of junk food and associated brands (e.g., fried chicken/KFC; burgers/McDonalds, etc.) and asked to select which categories they currently enjoy consuming. This selection was used to create a post hoc subgroup of participants who happened to be randomly exposed to a junk food advertisement that aligned with their preferences.

#### 2.2.3 Self‐Control

Four restraint items from the Brief Self Control Scale [27] estimated self‐control (e.g., I am good at resisting temptation). Responses were made on a 5‐point scale, anchored from [1] *not at all like me* to [5] *very much like me*. Item responses were averaged to calculate a total score. The restraint scale demonstrated good internal consistency (*α* = 0.79). This scale has been used previously to explore the role of psychological traits for susceptibility to junk food advertising [8].

#### 2.2.4 Impulsivity

Eight items from the brief Barrat Impulsivity Scale (BIS; [28]) estimated impulsiveness (e.g., I do things without thinking). Responses were made on a 4‐point scale anchored from [1] *Rarely/Never* to [4] *Almost Always/Always*. Item responses were averaged to calculate a total score. The BIS demonstrated good internal consistency (*α* = 0.80). This scale has been used previously to explore the role of psychological traits for susceptibility to junk food advertising [8].

#### 2.2.5 Junk Food and Anti‐Junk Food Advertisements

Eleven unique junk food advertisements sourced from video recordings of free‐to‐air national sports matches broadcast in Western Australia comprised the sample of stimuli, including four different McDonalds advertisements, two different Kentucky Fried Chicken (KFC) advertisements, two Uber Eats chocolate sauce with ice cream advertisements, one Subway hotdog advertisement, one Heinz hot chips/sausage roll/meat pie and sauce advertisement, and one Pringles advertisement. Note that 15‐s junk advertisements (*N* = 7) were collapsed into one condition and 30‐s junk advertisements (*N* = 4) were collapsed into one condition.

Three anti‐junk food advertisements were also sourced from the ‘Live Lighter’ YouTube channel which warned of the harms of junk food. One of these was a 30‐s video which depicted cancer growing on internal organs before showing a person eating a beef and cheese burger accompanied by a voiceover concluding with the message ‘A common reason for excess body fat is eating too much junk food, reduce the junk, reduce your cancer risk’. Two 15‐s advertisements were also obtained, both also depicting, an albeit shorter version of, cancer growing on internal organs. However, the two shorter advertisements differed in their ending. One ended in the same concluding voiceover as the 30‐s advertisement, but the other instead showed a person eating a healthy sandwich accompanied by a voiceover concluding ‘You might have tried to cut down on junk food before, but why not try again, healthy options are at hand, reduce the junk, reduce your cancer risk’. With these two 15‐s versions differing in the framing of their message, we analysed these conditions separately to determine if the framing impacted craving or consumption intentions (C: criticising junk food; E: encouraging healthy food).

Two 15‐s neutral advertisements unrelated to food were randomly selected for comparative purposes: a superannuation advertisement and a tyre advertisement from two well‐known brands. To facilitate viewership for participants during the online experiment, all advertisements were individually privately hosted on YouTube. Links to all videos are available in the Supporting Information.

### 2.3 Procedure

The Qualtrics survey platform [29] deployed the experiment, presented to participants as a study on attitudes towards food advertising and involving some brief questions about their current attitudes towards food followed by a short video clip taken from a TV broadcast. Participants provided consent and completed baseline pre‐exposure measures of craving and consumption intentions before being randomly allocated to one of the following six video conditions: (1) a neutral advertisement, (2) a 30‐s anti‐junk food advertisement, (3 and 4) a 15‐s anti‐junk food (C or E) advertisement, (5) a 30‐s junk food advertisement or a (6) 15‐s junk food advertisement. Participants (*N* = 23) taking longer than 60 s to progress from the commencement of the video to commencement of the post‐exposure measures were excluded to mitigate the potential impact of distraction. After post‐exposure measures, participants completed the HUEBS, restraint items and the BIS in a randomised order, followed by demographic questions, including height and weight. University‐recruited participants earned course credit and Qualtrics panel participants received points, gift cards or monetary remuneration. The research was approved by the university human research ethics committee (Ref: 2022‐03789‐HOLLETT). All participants provided informed consent prior to the collection of data.

### 2.4 Research Design and Analysis

For the experimental analyses, mixed‐model ANOVAs tested two independent variables, a within‐subjects factor (Video Exposure: Pre; Post) and a between‐subjects factor (Exposure Type: Neutral; 30 s Anti‐Junk; 15 s Anti‐Junk C; 15 s Anti‐Junk E; 30 s Junk; 15 s Junk), on the dependent variables of junk food craving and consumption intentions. These analyses were repeated separately for participants classified as ‘normal weight’ (BMI: 18.5–24.99 kg/m^2^) and those classified as ‘overweight’ (BMI: > 24.99 kg/m^2^) or those classified as having obesity (BMI: > 29.99 kg/m^2^) as defined by the Centres for Disease Control and Prevention [30]. That is, the ‘overweight’ participants and those with obesity were analysed together and referred to as the OW/OB group. ‘Underweight’ participants (BMI: < 18.5 kg/m^2^) were excluded as there were insufficient participants representing this subgroup to draw confident conclusions (*N* = 22). We also examined a third post hoc ‘matched preferences’ subgroup comprised of participants whose junk food preferences happened to align with the junk food they were randomly exposed to (*N* = 99). All follow up tests used a *p* = 0.01 threshold for significance to protect against type I error.

For the correlational analyses, Pearson correlations were performed between experimental measures (craving; consumption intentions) and trait measures (HUEBS; restraint; BIS). We interpreted *d* values (0.20, 0.40, 0.60) and *r* values (0.10, 0.20, 0.30) as small, typical and relatively large (respectively) [31]. All variables were normally distributed (skew < |2.00|; kurtosis < |3.00|). All analyses were conducted using the Statistical Package for Social Sciences (SPSS), version 29. The data have been provided in the Supporting Information.

## 3 Results

### 3.1 Participant Characteristics

Participants identified as men (49%), women (49%) and other (2%). Participants were aged between 18–90 years (M = 38.60, SD = 17.87) and were mostly Caucasian (78%), followed by Asian (11%), Aboriginal or Torres Strait islander (1%), African (2%) or mixed/other (8%). The average BMI was 27.32 (SD = 6.76) with 45% (*N* = 226) participants classified as normal weight and 55% (*N* = 279) classified as overweight or having obesity (OW/OB group).

### 3.2 Craving and Consumption Intentions in the Normal BMI Group

**FIGURE 1:**
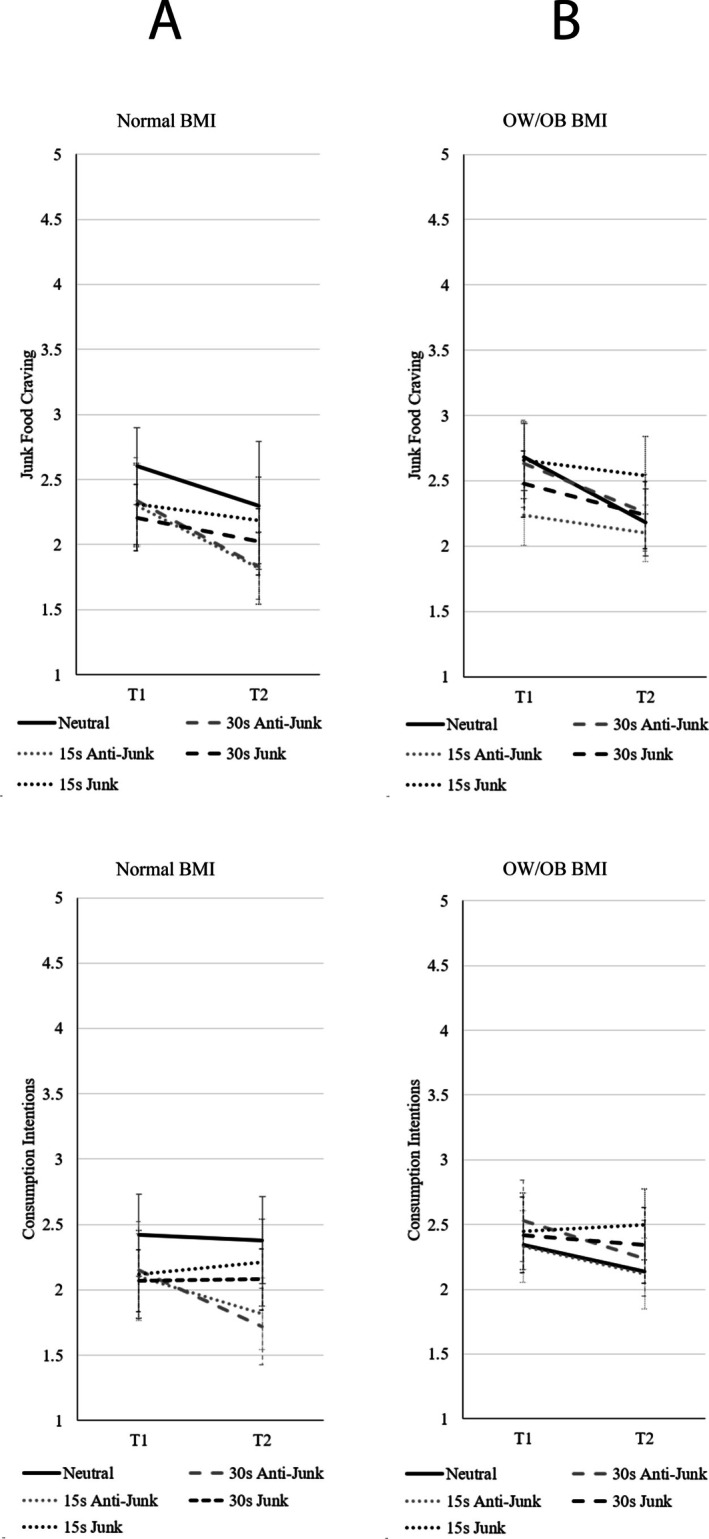
Means and 95% confidence intervals for junk food craving and consumption intentions across exposure types, separated for participants with (A) normal BMI and (B) those with BMI classified as overweight or having obesity (OW/OB).

**FIGURE 2:**
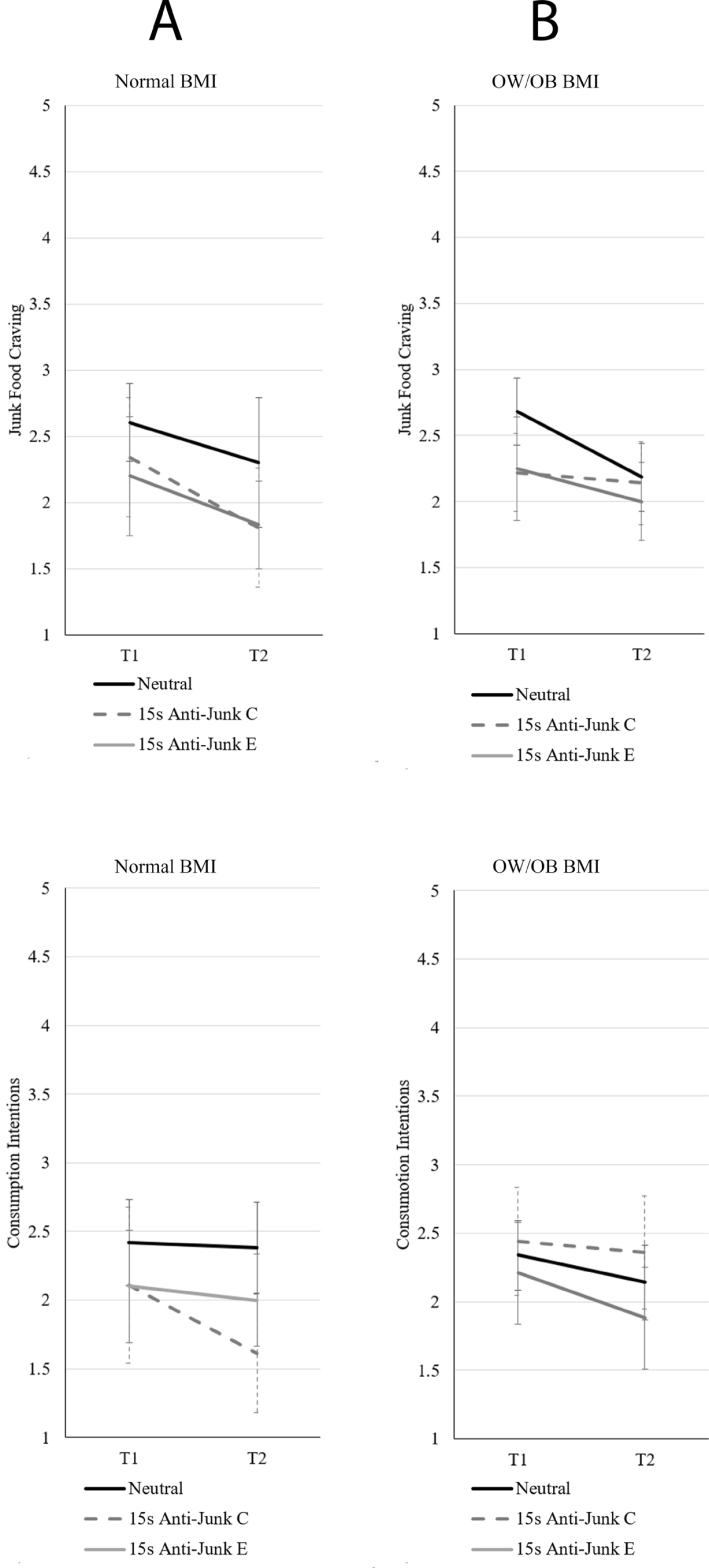
Means and 95% confidence intervals for junk food craving and consumption intentions for anti‐junk messages criticising junk food (C) and encouraging healthy food (E), separated for participants with (A) normal BMI and (B) those with BMI classified as overweight or having obesity (OW/OB).

For the craving score, there was a main effect of video exposure, *F*(1, 220) = 58.63, *p* < 0.001, η_p_^2^ = 0.21, but not exposure type, *F*(6, 220) = 1.16, *p* = 0.330, η_p_^2^ = 0.03. However, there was a two‐way interaction, *F*(5, 220) = 2.69, *p* = 0.022, η_p_^2^ = 0.06, indicating the changes in craving were dependent on the exposure type. Paired samples *t*‐tests comparing pre and post exposure for each exposure type revealed there were significant reductions in junk food craving following exposure to the neutral (*p* < 0.001, *d* = 0.69), 30s anti‐junk (*p* < 0.001, *d* = 0.56), 15 s anti‐junk C (*p* < 0.001, *d* = 1.12), and 30 s junk (*p* = 0.003, *d* = 0.41) advertisements, but no significant change (when using a corrected alpha) in craving following exposure to the 15 s anti‐junk E (*p* = 0.029, *d* = 0.51) and 15 s junk (*p* = 0.184, *d* = 0.23) advertisements. See Figure 1 for means and confidence intervals. Note that, for clarity, the 15‐s anti‐junk food video conditions have been collapsed in Figure 1 but expanded in Figure 2 to illustrate the relative impact of length and framing effects of the anti‐junk advertisements.

For consumption intentions, there was a main effect of video exposure, *F*(1, 220) = 10.36, *p* = 0.001, η_p_^2^ = 0.04, but not exposure type, *F*(5, 220) = 1.39, *p* = 0.230, η_p_^2^ = 0.03. However, there was a two‐way interaction, *F*(5, 220) = 4.27, *p* < 0.001, η_p_^2^ = 0.09, indicating that the changes in intentions were dependent on the exposure type. Paired samples *t*‐tests comparing pre and post exposure for each exposure type revealed there were significant reductions in consumption intentions following exposure to the 30 s anti‐junk (*p* < 0.001, *d* = 0.52) and the 15 s anti‐junk C (*p* = 0.003, *d* = 0.81) advertisements, but no significant change in consumption intentions following exposure to the neutral (*p* = 0.710, *d* = 0.05), 15 s anti‐junk E (*p* = 0.329, *d* = 0.22), 30 s junk (*p* = 0.829, *d* = −0.03) and 15 s junk (*p* = 0.414, *d* = −0.14) advertisements. See Figures 1 and 2 for means and confidence intervals. As neither craving nor consumption intentions significantly increased across the junk food exposure types, hypotheses 1a,b were not supported in the normal BMI group. However, as there was a significant decrease in junk food craving and consumption intentions following exposure to some of the anti‐junk food advertisements, hypotheses 2a,b were supported in the normal BMI group.

### 3.3 Craving and Consumption Intentions in the OW/OB BMI Group

For the craving score, there was a main effect of video exposure, *F*(1, 273) = 42.83, *p* < 0.001, η_p_^2^ = 0.14, but not exposure type, *F*(5, 273) = 1.08, *p* = 0.370, η_p_^2^ = 0.02. However, there was a two‐way interaction, *F*(5, 273) = 3.24, *p* = 0.007, η_p_^2^ = 0.06, indicating that the changes in craving were dependent on the exposure type. Paired samples *t*‐tests comparing pre and post‐exposure for each exposure type revealed there were significant reductions in junk food craving following exposure to the neutral (*p* < 0.001, *d* = 0.75), 30 s anti‐junk (*p* < 0.001, *d* = 0.60) and 15 s anti‐junk E (*p* = 0.009, *d* = 0.58) advertisements, but not the 15 s anti‐junk C (*p* = 0.419, *d* = 0.16), 30 s junk (*p* = 0.010, *d* = 0.38) or 15 s junk (*p* = 0.154, *d* = 0.19) advertisements.

For consumption intentions, there was a main effect of video exposure, *F*(1, 273) = 8.84, *p* = 0.003, η_p_^2^ = 0.03, but not exposure type, *F*(5, 273) = 0.81, *p* = 0.540, η_p_^2^ = 0.01 and no two‐way interaction, *F*(5, 273) = 1.51, *p* = 0.187, η_p_^2^ = 0.03. To follow up the main effect, paired samples *t*‐tests comparing pre and post exposure for each exposure type were conducted. There was a significant reduction in consumption intentions following exposure to the 30 s anti‐junk (*p* = 0.008, *d* = 0.35) advertisement, but no significant change in consumption intentions following exposure to the neutral (*p* = 0.013, *d* = 0.34), 15 s anti‐junk C (*p* = 0.627, *d* = 0.01), 15 s anti‐junk E (*p* = 0.043, *d* = 0.44), 30 s junk (*p* = 0.584, *d* = 0.08) or 15 s junk (*p* = 0.616, *d* = −0.07) advertisements. See Figures 1 and 2 for the means and confidence intervals. As neither craving nor consumption intentions significantly increased across the junk food exposure types, hypotheses 1a,b were not supported in the OW/OW BMI group. However, as there was a significant decrease in junk food craving and consumption intentions following exposure to some of the anti‐junk food advertisements, hypotheses 3a,b were supported in the OW/OB BMI group.

Given that neither group showed increases in craving or consumption intentions following junk food exposure, hypothesis 1c was also not supported. Following the 30 s anti‐junk advertisement, there was a similar reduction in craving for each BMI group; however, the reduction in consumption intentions was stronger for the normal compared to the OW/OB BMI group, thus hypothesis 2c was supported when participants were exposed to a 30 s anti‐junk advertisement.

### 3.4 Matched Junk Food Preference Subgroup Analyses

**FIGURE 3:**
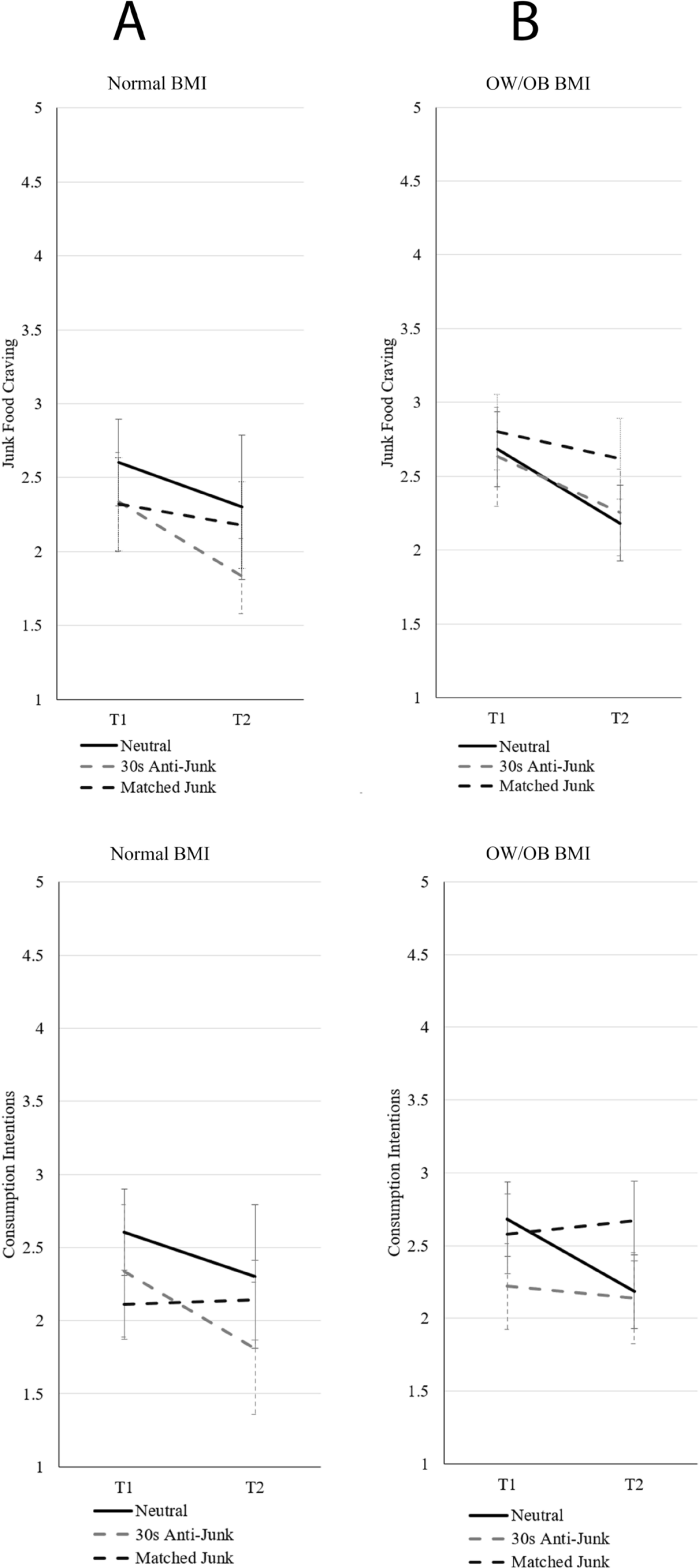
Means and 95% confidence intervals for junk food craving and consumption intentions for matched junk food preference subgroup with neutral and anti‐junk conditions for relative comparison, separated for participants with (A) normal BMI and (B) those with BMI classified as overweight or having obesity (OW/OB).

For the normal BMI subgroup (*N* = 44), paired samples *t*‐tests comparing pre and post exposure measures revealed there were no significant increases in craving (*p* = 0.041, *d* = 0.32) or consumption intentions (*p* = 0.785, *d* = −0.04) when exposed to junk foods which matched participants' preferences. For the OW/OB BMI subgroup, paired samples *t*‐tests comparing pre and post exposure measures revealed there were no significant increases in craving (*p* = 0.045, *d* = 0.28) or consumption intentions (*p* = 0.505, *d* = −0.09) when exposed to junk foods which matched participants' preferences. As neither craving nor consumption intentions increased in these subgroups, hypotheses 3a,b were not supported. See Figure 3 for means and confidence intervals.

### 3.5 Correlational Analyses

As can be seen in Table 1, there were significant correlations between healthy eating, unhealthy eating, restraint, impulsivity and the experimental variables in at least one condition. The correlations were generally larger for unhealthy eating compared to healthy eating across the conditions, and for restraint compared to impulsivity, but largely similar pre and post exposure. Only the correlation between craving and restraint in the 15 s junk condition was statistically stronger post exposure compared to pre‐exposure (confirmed via Fisher's *Z* test, *p* = 0.021), which was supportive of hypothesis 4c. This suggests that participants who generally exhibit lower restraint are more susceptible to higher junk food craving following junk food exposure. No other correlations were statistically stronger following junk food exposure for any other condition.

**TABLE 1:** Correlations between experimental variables and individual differences measures and descriptive statistics, separated across video conditions.

	Correlations				Descriptives	
	HUEBS (H)	HUEBS (U)	Restraint	BIS	Mean	SD
Neutral advertisements (*N* = 106)						
Craving pre‐exposure	**−0.40**	**0.54**	**−0.61**	**0.43**	2.65	1.02
Craving post‐exposure	**−0.29**	**0.49**	**−0.54**	**0.36**	2.24	1.01
Intentions pre‐exposure	**−0.34**	**0.49**	**−0.53**	**0.34**	2.38	1.02
Intentions post‐exposure	**−0.26**	**0.38**	**−0.47**	**0.32**	2.25	1.11
30 s anti‐junk advertisement (*N* = 108)						
Craving pre‐exposure	**−0.26**	**0.42**	**−0.32**	**0.41**	2.51	1.25
Craving post‐exposure	**−0.27**	**0.41**	**−0.29**	**0.26**	2.08	1.10
Intentions pre‐exposure	0.02	**0.49**	−0.04	**0.24**	2.37	1.25
Intentions post‐exposure	−0.16	**0.41**	−0.08	0.10	2.02	1.13
15 s anti‐junk advertisements (*N* = 88)						
Craving pre‐exposure	**−0.38**	**0.38**	**−0.36**	0.03	2.25	0.92
Craving post‐exposure	**−0.36**	**0.42**	**−0.32**	**0.22**	1.95	0.82
Intentions pre‐exposure	−0.19	**0.41**	−0.19	0.01	2.23	1.01
Intentions post‐exposure	−0.20	**0.41**	**−0.25**	0.15	1.99	0.95
30 s junk advertisements (*N* = 109)						
Craving pre‐exposure	**−0.19**	**0.40**	**−0.32**	**0.38**	2.33	0.98
Craving post‐exposure	**−0.20**	**0.33**	**−0.31**	**0.35**	2.12	0.95
Intentions pre‐exposure	−0.09	**0.25**	−0.14	**0.31**	2.23	1.01
Intentions post‐exposure	−0.05	**0.33**	−0.16	**0.33**	2.2	1.01
15 s junk advertisements (*N* = 94)						
Craving pre‐exposure	**−0.42**	**0.46**	**−0.40**	**0.39**	2.53	1.11
Craving post‐exposure	**−0.43**	**0.50**	**−0.50**	**0.42**	2.41	1.13
Intentions pre‐exposure	**−0.44**	**0.54**	**−0.45**	**−0.30**	2.33	1.11
Intentions post‐exposure	**−0.34**	**0.56**	**−0.40**	**−0.36**	2.39	1.04
Mean	3.83	2.99	2.84	2.14		
SD	0.92	1.15	0.91	0.52		

*Note:* Significant (*p* < 0.05) correlations in boldface. Exact *p*‐values for each correlation have been reported in the Supporting Information. Note that an additional table separating the descriptive statistics across all BMI groups and the two 15 s anti‐junk advertisements (C and E) has been provided in the Supporting Information.

Abbreviations: BIS, Barratt Impulsivity Scale (brief); BMI, body mass index; HUEBS, Healthy (H) and Unhealthy (U) Eating Behaviour Scales.

## 4 Discussion

The present study explored consumption inclination responses to various length junk and anti‐junk food advertisements among those with normal and higher than normal BMI classifications. Regardless of length, none of the junk food advertisements significantly increased craving or consumption intentions, which is consistent with prior meta‐analytic and experimental research in adults [7, 8, 32]. These effects remained consistent even when analysing a subgroup of participants who were exposed to a junk food they usually enjoy consuming. As such, we conclude that a single exposure to a 15 or 30 s junk food advertisement did not increase immediate inclinations to consume junk food. However, it must be recognised that repeated exposure to junk food advertisements is likely to be considerably more effective for enhancing brand loyalty and adversely affecting longer term junk food consumption patterns [32, 33]. Furthermore, junk food advertisements contribute to the normalisation of junk food as a regular part of a diet, which can be particularly threatening to the health of vulnerable individuals in the adult population and among adolescents and children [33, 34]. Indeed, more experimental work on multiple exposures in adults is needed to clarify whether stronger effects might be observed on inclinations to consume junk food and normalising unhealthy consumption patterns, as has been found in children [7, 33].

Importantly, anti‐junk food advertisements did successfully decrease junk food inclinations in both BMI groups, although interesting nuances emerged when separately analysing advertisements of different length and framing. Specifically, while both BMI groups responded similarly to the 30‐s anti‐junk food advertisement, normal BMI group participants responded more strongly to the 15‐s junk food critical advertisement, whereas the OW/OB BMI group responded more strongly to the 15 s health food encouragement advertisement. These results confirm that 15‐s advertisements may offer greater efficiency whilst allowing more impact in the same timeframe as a longer advertisement. That is, for every 30‐s Live Lighter advertisement, two 15‐s advertisements could be aired with different framing to more comprehensively target separate audiences.

These anti‐junk food advertisement findings are consistent with prior experimental research examining the impact of health messages [8, 32] and complement several cohort studies on the Live Lighter campaign showing general reductions in junk food consumption, as well as increases in awareness of unhealthy eating and the importance of physical activity [35, 36, 37, 38, 39]. However, our data inform specific mitigation strategies related to length and framing of advertisements which could maximise the cost–benefit of health messaging.

Given the different effects observed across normal and OW/OB BMI groups and the strong associations between advertisement inclination responses and eating habits, we strongly encourage public health researchers to account for individual differences in health status and psychological traits when evaluating the impact of health promotion strategies. While public health campaigns which attempt a one‐size‐fits‐all approach may offer some convenience, our data support the necessity to continue developing multiple messages which differ in their framing to cater for heterogeneous audiences. Importantly, our findings demonstrate short‐term changes in inclinations following health messaging which justifies further investment in public health messages aiming to mitigate junk food consumption. Recent content analysis research on free‐to‐air advertising suggests that junk food advertising outweighs anti‐junk food advertising almost 50 to 1 [8]. Given that a higher frequency of health messaging is likely necessary to counteract the dominance of junk food advertising, the present study provides compelling evidence supporting the efficacy of more‐frequently‐aired 15‐s advertisements rather than less‐frequently‐aired 30‐s advertisements. Junk food advertising restrictions, coupled with frequent health messaging, is essential to mitigate corporate marketing power and consistent with international policy recommendations by the World Health Organisation on food marketing to children [40]. Importantly, evidence from multiple recent Australian studies indicate that young adults are especially targeted by junk food advertising and 40%–62% of adults report attitudes supportive of junk food advertising restrictions [41, 42].

### 4.1 Limitations and Future Directions

Despite the large sample and experimental design, there are limitations to the conclusions of the present study. Firstly, the data do not inform an understanding of repeated exposures and longer‐term effects of junk food advertisements on junk food consumption inclinations and dietary patterns. Secondly, the experimental exposure occurred online without high levels of control and relied upon the self‐report of variables, some of which could be objectively measured, such as height and weight. Furthermore, additional diagnostic criteria (e.g., waist circumference) would be desirable to properly classify individuals living with obesity [43]. While this exposure paradigm aligns with how many video advertisements are now experienced in the real world, we recognise that various sources of measurement error may have obscured some experimental effects. Finally, our study findings cannot be generalised to other contexts or advertising formats such as banners and dynamic images encountered on social media and video streaming platforms. Further work is needed to understand the impact of various forms of junk and anti‐junk advertising media across different platforms, preferably under both controlled and uncontrolled conditions.

## 5 Conclusions

In conclusion, we found that a single exposure to a randomly selected junk food advertisement did not elicit a statistically significant immediate inclination to consume junk food among adults, even when participants reported enjoying the junk food they were exposed to. However, a brief single exposure to an anti‐junk food advertisement was shown to reduce junk food craving and consumption intentions in both normal and OW/OB BMI groups. While exposure to a 30‐s anti‐junk food advertisement similarly reduced junk food craving in both groups, a 15‐s advertisement critiquing junk was more effective for normal BMI participants on average, and a 15‐s advertisement encouraging healthy food was more effective in reducing inclinations for OW/OB BMI participants on average. Thus, brevity and targeted message framing may deliver optimal mitigation potential for reducing junk food consumption among Australian adults.

## Funding

This work was supported by the Edith Cowan University.

## Ethics Statement

The data collection procedures reported in this manuscript were approved by the Edith Cowan University Ethics Committee.

## Conflicts of Interest

The authors declare no conflicts of interest.

## Supporting information


**Table S1:** Descriptive statistics for all outcome measures and trait measures separated by BMI group.
**Table S2:** Correlation *p*‐values between experimental variables and individual differences measures and descriptive statistics, separated across video conditions

**Data S1:** Youtube links to video stimuli.

**Data S2:** Supporting Information.

## Data Availability

The data that supports the findings of this study are available in the Supporting Information of this article.

## Endnotes

^1^
Qualtrics is a market research service provider: https://www.qualtrics.com/au/research‐services/online‐sample/.

^2^
We excluded one item related to alcohol as it referred to guidelines not endorsed in Australia.
