# Supplementary material for: Length and Framing of Anti‐Junk Food Ads Impact Inclinations to Consume Junk Food Among Normal Weight, Overweight, and Adults With Obesity
Source: Health Promot J Austr. 2026 Feb 2;37(2):e70159. doi: 10.1002/hpja.70159 (PMC12865134; doi:10.1002/hpja.70159)
Supplement: Supplementary file 1 — Table S1: Descriptive statistics for all outcome measures and trait measures separated by BMI group. Table S2: Correlation p‐values between experimental variables and individual differences measures and descriptive statistics, separated across video conditions [file HPJA-37-0-s002.docx]

**Supplementary Table 1.**

*Descriptive Statistics for all Outcome Measures and Trait Measures Separated by BMI Group*

|  | Normal BMI  (*N* = 226) | | Overweight BMI  (*N* = 158) | | Obesity BMI (*N* = 121) | | Overall Sample (*N* = 505) | |
| --- | --- | --- | --- | --- | --- | --- | --- | --- |
|  | *M* | *SD* | *M* | *SD* | *M* | *SD* | *M* | *SD* |
| Neutral Advertisements (*N* = 106) |  |  |  |  |  |  |  |  |
| Craving pre-exposure | 2.60 | 1.03 | 2.45 | 0.91 | 3.10 | 1.10 | 2.65 | 1.02 |
| Craving post-exposure | 2.30 | 1.04 | 1.95 | 0.90 | 2.60 | 1.06 | 2.24 | 1.01 |
| Intentions pre-exposure | 2.42 | 1.09 | 2.14 | 0.86 | 2.71 | 1.06 | 2.38 | 1.02 |
| Intentions post-exposure | 2.38 | 1.20 | 1.95 | 0.97 | 2.48 | 1.08 | 2.25 | 1.11 |
| 30s Anti-Junk Advertisement (*N* = 108) |  |  |  |  |  |  |  |  |
| Craving pre-exposure | 2.33 | 1.13 | 2.44 | 1.31 | 2.94 | 1.30 | 2.51 | 1.25 |
| Craving post-exposure | 1.83 | 0.88 | 2.15 | 1.17 | 2.42 | 1.29 | 2.08 | 1.10 |
| Intentions pre-exposure | 2.15 | 1.26 | 2.47 | 1.33 | 2.63 | 1.06 | 2.37 | 1.25 |
| Intentions post-exposure | 1.72 | 1.03 | 2.26 | 1.20 | 2.21 | 1.10 | 2.02 | 1.13 |
| 15s Anti-Junk Advertisement C (*N* = 45) |  |  |  |  |  |  |  |  |
| Craving pre-exposure | 2.33 | 0.99 | 2.17 | 0.79 | 2.28 | 0.69 | 2.27 | 0.84 |
| Craving post-exposure | 1.78 | 0.98 | 2.26 | 0.85 | 2.02 | 0.78 | 1.99 | 0.90 |
| Intentions pre-exposure | 2.11 | 1.23 | 2.46 | 0.97 | 2.42 | 1.08 | 2.30 | 1.10 |
| Intentions post-exposure | 1.61 | 0.92 | 2.38 | 0.96 | 2.33 | 1.15 | 2.05 | 1.05 |
| 15s Anti-Junk Advertisement E (*N* = 43) |  |  |  |  |  |  |  |  |
| Craving pre-exposure | 2.20 | 1.07 | 2.12 | 0.71 | 2.34 | 1.14 | 2.23 | 1.01 |
| Craving post-exposure | 1.83 | 0.79 | 1.92 | 0.63 | 2.06 | 0.80 | 1.92 | 0.75 |
| Intentions pre-exposure | 2.10 | 0.94 | 2.10 | 0.74 | 2.29 | 1.07 | 2.16 | 0.93 |
| Intentions post-exposure | 2.00 | 0.77 | 1.90 | 1.10 | 1.86 | 0.86 | 1.93 | 0.86 |
| 30s Junk Advertisements (*N* = 109) |  |  |  |  |  |  |  |  |
| Craving pre-exposure | 2.21 | 1.02 | 2.54 | 0.91 | 2.38 | 0.94 | 2.33 | 0.98 |
| Craving post-exposure | 2.02 | 0.96 | 2.35 | 0.91 | 2.04 | 0.94 | 2.12 | 0.95 |
| Intentions pre-exposure | 2.07 | 0.91 | 2.42 | 1.09 | 2.42 | 1.12 | 2.23 | 1.01 |
| Intentions post-exposure | 2.08 | 0.93 | 2.48 | 1.06 | 2.11 | 1.10 | 2.20 | 1.01 |
| 15s Junk Advertisements (*N* = 94) |  |  |  |  |  |  |  |  |
| Craving pre-exposure | 2.31 | 0.92 | 2.15 | 1.02 | 3.14 | 1.15 | 2.53 | 1.11 |
| Craving post-exposure | 2.19 | 1.01 | 2.05 | 1.07 | 3.01 | 1.09 | 2.41 | 1.13 |
| Intentions pre-exposure | 2.12 | 1.01 | 1.90 | 1.01 | 2.97 | 1.05 | 2.33 | 1.11 |
| Intentions post-exposure | 2.21 | 0.98 | 2.07 | 1.00 | 2.90 | 0.98 | 2.39 | 1.04 |
| Matched Junk Advertisements (*N* = 99) | (*N* = 44) | | (*N* = 31) | | (*N* = 24) | |  | |
| Craving pre-exposure | 2.32 | 1.03 | 2.68 | 0.95 | 2.97 | 1.01 | 2.59 | 1.03 |
| Craving post-exposure | 2.18 | 1.00 | 2.50 | 1.02 | 2.78 | 1.06 | 2.42 | 1.04 |
| Intentions pre-exposure | 2.11 | 0.81 | 2.45 | 1.06 | 2.75 | 0.99 | 2.37 | 0.97 |
| Intentions post-exposure | 2.14 | 0.90 | 2.58 | 1.06 | 2.79 | 1.02 | 2.43 | 1.01 |
| Trait Measures |  |  |  |  |  |  |  |  |
| HUEBS Healthy Item Score | 3.91 | 0.95 | 3.92 | 0.81 | 3.57 | 0.96 | 3.83 | 0.92 |
| HUEBS Unhealthy Item Score | 2.89 | 1.16 | 3.08 | 1.10 | 3.05 | 1.19 | 2.99 | 1.15 |
| Restraint | 2.92 | 0.91 | 2.92 | 0.88 | 2.60 | 0.92 | 2.84 | 0.91 |
| BIS | 2.13 | 0.51 | 2.11 | 0.52 | 2.21 | 0.52 | 2.14 | 0.52 |

*^Note^*^. BMI = Body Mass Index; HUEBS = Healthy and Unhealthy Eating Behavior Scale; BIS = Barratt Impulsivity Scale (brief); C = criticizes junk food; E = encourages healthy food^

**Supplementary Table 2.**

*Correlation p values between Experimental Variables and Individual Differences Measures and Descriptive Statistics, Separated Across Video Conditions*

|  | Correlation *p* values | | | |  | Descriptives | |
| --- | --- | --- | --- | --- | --- | --- | --- |
|  | HUEBS (H) | HUEBS (U) | Restraint | BIS |  | Mean | SD |
| Neutral Advertisements (*N* = 106) |  |  |  |  |  |  |  |
| Craving pre-exposure | **<0.001** | **<0.001** | **<0.001** | **<0.001** |  | 2.65 | 1.02 |
| Craving post-exposure | **<0.001** | **<0.001** | **<0.001** | **<0.001** |  | 2.24 | 1.01 |
| Intentions pre-exposure | **<0.001** | **<0.001** | **<0.001** | **<0.001** |  | 2.38 | 1.02 |
| Intentions post-exposure | **0.007** | **<0.001** | **<0.001** | **<0.001** |  | 2.25 | 1.11 |
|  |  |  |  |  |  |  |  |
| 30s Anti-Junk Advertisement (*N* = 108) |  |  |  |  |  |  |  |
| Craving pre-exposure | **0.006** | **<0.001** | **<0.001** | **<0.001** |  | 2.51 | 1.25 |
| Craving post-exposure | **0.005** | **<0.001** | **0.003** | **0.008** |  | 2.08 | 1.10 |
| Intentions pre-exposure | 0.849 | **<0.001** | 0.646 | **0.011** |  | 2.37 | 1.25 |
| Intentions post-exposure | 0.108 | **<0.001** | 0.428 | 0.282 |  | 2.02 | 1.13 |
|  |  |  |  |  |  |  |  |
| 15s Anti-Junk Advertisements (*N* = 88) |  |  |  |  |  |  |  |
| Craving pre-exposure | **<0.001** | **<0.001** | **<0.001** | 0.747 |  | 2.25 | 0.92 |
| Craving post-exposure | **<0.001** | **<0.001** | **0.002** | **0.038** |  | 1.95 | 0.82 |
| Intentions pre-exposure | 0.840 | **<0.001** | 0.074 | 0.904 |  | 2.23 | 1.01 |
| Intentions post-exposure | 0.057 | **<0.001** | **0.019** | 0.154 |  | 1.99 | 0.95 |
|  |  |  |  |  |  |  |  |
| 30s Junk Advertisements (*N* = 109) |  |  |  |  |  |  |  |
| Craving pre-exposure | **0.050** | **<0.001** | **<0.001** | **<0.001** |  | 2.33 | 0.98 |
| Craving post-exposure | **0.036** | **<0.001** | **0.001** | **<0.001** |  | 2.12 | 0.95 |
| Intentions pre-exposure | 0.369 | **0.009** | 0.152 | **0.001** |  | 2.23 | 1.01 |
| Intentions post-exposure | 0.618 | **<0.001** | 0.100 | **<0.001** |  | 2.2 | 1.01 |
|  |  |  |  |  |  |  |  |
| 15s Junk Advertisements (*N* = 94) |  |  |  |  |  |  |  |
| Craving pre-exposure | **<0.001** | **<0.001** | **<0.001** | **<0.001** |  | 2.53 | 1.11 |
| Craving post-exposure | **<0.001** | **<0.001** | **<0.001** | **<0.001** |  | 2.41 | 1.13 |
| Intentions pre-exposure | **<0.001** | **<0.001** | **<0.001** | **0.003** |  | 2.33 | 1.11 |
| Intentions post-exposure | **<0.001** | **<0.001** | **<0.001** | **<0.001** |  | 2.39 | 1.04 |
|  |  |  |  |  |  |  |  |
| Mean | 3.83 | 2.99 | 2.84 | 2.14 |  |  |  |
| SD | 0.92 | 1.15 | 0.91 | 0.52 |  |  |  |

*^Note^*^. BMI = Body Mass Index; HUEBS = Healthy (H) and Unhealthy (U) Eating Behavior Scales; BIS = Barratt Impulsivity Scale (brief)^
