# Supplementary material for: Length and Framing of Anti‐Junk Food Ads Impact Inclinations to Consume Junk Food Among Normal Weight, Overweight, and Adults With Obesity
Source: Health Promot J Austr. 2026 Feb 2;37(2):e70159. doi: 10.1002/hpja.70159 (PMC12865134; doi:10.1002/hpja.70159)
Supplement: Supplementary file 2 — Data S1: Youtube links to video stimuli. [file HPJA-37-0-s001.docx]

**Youtube Links to Video Stimuli**

- Live Lighter 30s – criticising junk food

<https://www.youtube.com/watch?v=qgq7P8zObzg>

- Live Lighter 15s – encouraging healthy food

<https://www.youtube.com/watch?v=BvRs_3gCjSE>

- Live Lighter 15s – criticising junk food

<https://www.youtube.com/watch?v=FHE3nJvvXM8>

- Neutral Ads

<https://www.youtube.com/watch?v=kXJ6llYy5R8>

<https://youtu.be/ux_hQPj31ms>

- Junk Food Ads

<https://www.youtube.com/watch?v=_AuNIeopT7Y>

<https://www.youtube.com/watch?v=vF3KGoMd0pw>

<https://www.youtube.com/watch?v=x0fXAUzwVPc>

<https://www.youtube.com/watch?v=1s4q7bnWttA>

<https://www.youtube.com/watch?v=k2qUSn9uW4c>

<https://www.youtube.com/watch?v=laLpbO2KhGw>

<https://www.youtube.com/watch?v=O__1jO3WqtY>

<https://www.youtube.com/watch?v=lMaHJKaxBak>

<https://www.youtube.com/watch?v=ZATiVnKWP0c>

<https://www.youtube.com/watch?v=SKvhxgQ-GwI>

<https://www.youtube.com/watch?v=XQcb3rP4GAQ>
